# Commentary: *In vivo* Neuroregeneration to Treat Ischemic Stroke Through NeuroD1 AAV-Based Gene Therapy in Adult Non-human Primates

**DOI:** 10.3389/fcell.2021.648020

**Published:** 2021-05-28

**Authors:** Xiaoqin Zhang, Fenghua Chen, Youcui Wang

**Affiliations:** Institute of Brain Science and Disease, Shandong Provincial Key Laboratory of Pathogenesis and Prevention of Neurological Disorders, Shandong Provincial Collaborative Innovation Center for Neurodegenerative Disorders, Qingdao University, Qingdao, China

**Keywords:** NeuroD1, astrocyte-to-neuron, non-human primates, *in vivo*, stroke

## Introduction

The loss of neurons is the most common cause of nervous system disorders, such as stroke, spinal cord injury (SCI), and neurodegenerative diseases including Alzheimer's disease (AD), Parkinson's disease (PD), and Huntington's disease (HD). Given that neurons cannot divide to regenerate themselves, together with the fact that only a small number of new neurons derived from external cell transplantation can survive *in vivo* (Goldman, 2016), few neuroregenerative strategies have succeeded in adult mammalian brains. In order to avoid the limitations of cell transplantation therapy, such as ethical issues and transplantation rejection (Chen et al., 2019), there are research groups that have developed a technology that allows reprogramming of astrocytes into functional new neurons *in situ*. For example, the single neural transcription factor NeuroD1-based gene therapy can successfully reprogram astrocytes into functional neurons in AD mouse brains (Guo et al., 2014), damaged spinal cord (Puls et al., 2020), and stroke mouse brains, as well as promote functional recovery in a mouse stroke model (Chen et al., 2020). Besides, astrocytes in the striatum of HD mice can be directly converted into GABAergic medium spinal neurons by co-overexpressing NeuroD1 and Dlx2, leading to motor function recovery and longer life spans (Wu et al., 2020). Three groups have shown that astrocytes also can be converted into dopaminergic neurons, which led to behavioral improvement in a mouse PD model by highly expressing three transcription factors, NeuroD1, Ascl1, and Lmx1a, and the microRNA, miR218, collectively referred to as NeAL218 (Fyfe, 2017; Rivetti di Val Cervo et al., 2017), or by depleting an RNA-binding protein called PTB (Arenas, 2020; Qian et al., 2020; Zhou et al., 2020). Additionally, in an adult rat model of SCI, astrocytes can be reprogrammed into neurons by overexpressing SOX2, leading to motor function recovery (Su et al., 2014), or into neural stem cells by highly expressing Zfp521 (Zarei-Kheirabadi et al., 2019). Notably, it has been confirmed that human astrocytes can be successfully converted into neurons or neuroblasts *in vitro* (Corti et al., 2012; Ghasemi-Kasman et al., 2015; Zhang et al., 2015; Li et al., 2016; Rivetti di Val Cervo et al., 2017; Yin et al., 2019; Qian et al., 2020). These studies provide a potential alternative approach to regenerate functional new neurons in the central nervous system of adult mammals by directly reprogramming glial cells *in situ* into neurons.

## Advantages of Neurod1-Mediated Astrocyte-To-Neuron Conversion For Evaluating Therapeutic Treatment After Stroke In Adult Non-Human Primates

Recently, Chen's research group firstly reported that an *in situ* neuronal regeneration approach using AAV NeuroD1-based gene therapy could repair damaged brains in adult non-human primates (NHPs) with ischemic stroke (Ge et al., 2020). These findings were based on earlier successful investigations *in vitro* and *in vivo* (Guo et al., 2014; Brulet et al., 2017; Chen et al., 2020; Wu et al., 2020; Zhang et al., 2020).

The results have shown that high expression of a neural transcription factor NeuroD1 in astrocytes can successfully reprogram nearly 90% of infected astrocytes into neurons in the monkey cortex following ischemic stroke, and those neurons could survive over 1 year. The neurons from NeuroD1-mediated astrocyte-to-neuron (AtN) conversion displayed Tbr1^+^ cortical neuron identity and were mostly located in the monkey gray matter (Ge et al., 2020), which is entirely consistent with earlier findings in rodent ischemic stroke models (Chen et al., 2020; Liu et al., 2020). Interestingly, the number and intrinsic proliferative property of astrocytes in the converted areas were not changed compared with those in the control side.

Moreover, it was extremely attractive that the NeuroD1-mediated AtN conversion not only significantly increased the cortical neuronal density, dendritic marker, and synaptic marker levels in the ischemic injured areas of the monkey cortex but also improved the microenvironment, including reducing the number of reactive microglia and macrophages (Ge et al., 2020). The improvement of the inflammatory microenvironment was beneficial for the survival of parvalbumin-positive GABAergic interneurons that were badly damaged by ischemic injury (Povysheva et al., 2019). Another interesting finding different from that in rodent animal models was that the expression of NeuroD1 was significantly decreased in newly regenerated neurons after 6 months of AAV injection, which may be beneficial in future clinical trials (Ge et al., 2020). Furthermore, this study reported that this treatment had a broad time window from 10 to 30 days following ischemic stroke, and the cell conversion effect is long-lasting, ranging from 2 months to 1 year after viral infection (Ge et al., 2020), suggesting that the NeuroD1-mediated AtN conversion therapy might be a potential approach for neural repair in NHP brain.

## Discussion

Many clinical trials of treatment for cerebral ischemia based on preclinical parameters obtained from rodent animal models have failed in the past (Turner et al., 2013), because the successful discoveries in rodent animal models may not be replicated in primates. The main reason for such problems is that the human brain is largely different from rodent brain, while the brains of large animal models, such as monkeys, are more similar to those of humans (Modo et al., 2018). Therefore, in order to confirm the validity of *in vivo* astrocyte conversion in human clinical trials, it is very important and necessary to first test such findings in adult NHP models, such as in monkeys. Previously, acute NHP stroke models often were used to test drug effects, and the drug administration time was typically within a few hours after stroke (Takamatsu et al., 2001; Cook and Tymianski, 2012, Cook et al., 2017). In this study, a focal stroke model with a very low mortality rate was employed through intracranial injection of endothelin-1 to induce blood vessel constriction in the motor cortex of rhesus macaque monkeys. This study demonstrated that *in situ* NeuroD1-mediated AtN conversion therapy had a broad time window from 10 to 30 days following ischemic stroke (Ge et al., 2020).

Nevertheless, in this study, some neuronal functions of converted neurons were not investigated, such as neuronal excitability, production, and release neurotransmitter. Furthermore, there is a question as to why and how NeuroD1-mediated AtN conversion could reduce the number of microglia and macrophages; unfortunately, the mechanisms were not investigated, and further studies will be required. However, there is mounting evidence that the expression of NeuroD1 is often high during early brain development and is lower in the adult brain (Pataskar et al., 2016); further studies are needed to clarify the mechanism of the significant decrease in NeuroD1 expression following AtN conversion in the monkey cortex over time. There was no evidence about a correlation with behavioral improvement in adult NHPs after stroke, and to our knowledge, neuronal recovery at the cellular level needs to be accompanied by behavioral improvement.

Taken together, this is the first report about successful application of NeuroD1-mediated AtN conversion technology in NHP models, which might fill some gaps between *in vivo* rodent models and *in vitro* human astrocyte culture models, as well as take an important step toward future clinical trials using such technology for nervous system diseases with neuronal loss.

## Author Contributions

XZ and FC wrote the first draft. YW reviewed and critiqued the manuscript. All authors contributed to the article and approved the submitted version.

## Conflict of Interest

The authors declare that the research was conducted in the absence of any commercial or financial relationships that could be construed as a potential conflict of interest.
